# Predicting the difficult laparoscopic cholecystectomy based on a preoperative scale

**DOI:** 10.1007/s13304-021-01216-y

**Published:** 2022-02-04

**Authors:** Camilo Ramírez-Giraldo, Kelly Alvarado-Valenzuela, Andrés Isaza-Restrepo, Jorge Navarro-Alean

**Affiliations:** 1 Hospital Universitario Mayor, Méderi, Bogotá, Colombia; 2 Universidad del Rosario, Carrera 24 #63C-69, Bogotá, Colombia

**Keywords:** Cholecystectomy, Operative difficulty, Laparoscopic

## Abstract

It is important to establish the difficulty of a cholecystectomy preoperatively to improve the outcomes. There are multiple risk factors for a difficult cholecystectomy that may depend on the patient, the disease, or extrinsic factors. The aim of this study is to evaluate the predictive capacity of a difficult cholecystectomy with a preoperative scale. A diagnostic trial study was designed to evaluate the performance of a scale to predict the difficulty of laparoscopic cholecystectomy, considering as a reference standard the intraoperative findings evaluated according to an intraoperative difficulty scale. A ROC curve was performed and used to estimate predictive value of the preoperative score to predict the difficulty of a cholecystectomy preoperatively. The ROC curve shows an area of 0.88 under the curve. The calculated ideal cutoff was 8, with a sensitivity, specificity, positive predictive value and negative predictive value of 75.15%, 88.31%, 87.32 and 76.83%, respectively. It was demonstrated that, as the difficulty predicted by the preoperative scale increases, the rate of conversion to open procedure, the rate of subtotal cholecystectomies, the rate of complication and the rate of a critical view of safety failed increase. We suggest implementing the preoperative scale in all patients who are planning laparoscopic cholecystectomy, considering it a simple and easy tool to perform. This to inform the patient, organize the surgery schedule, select personnel, request support and have adequate pre-operative planning.

**Graphical abstract:**

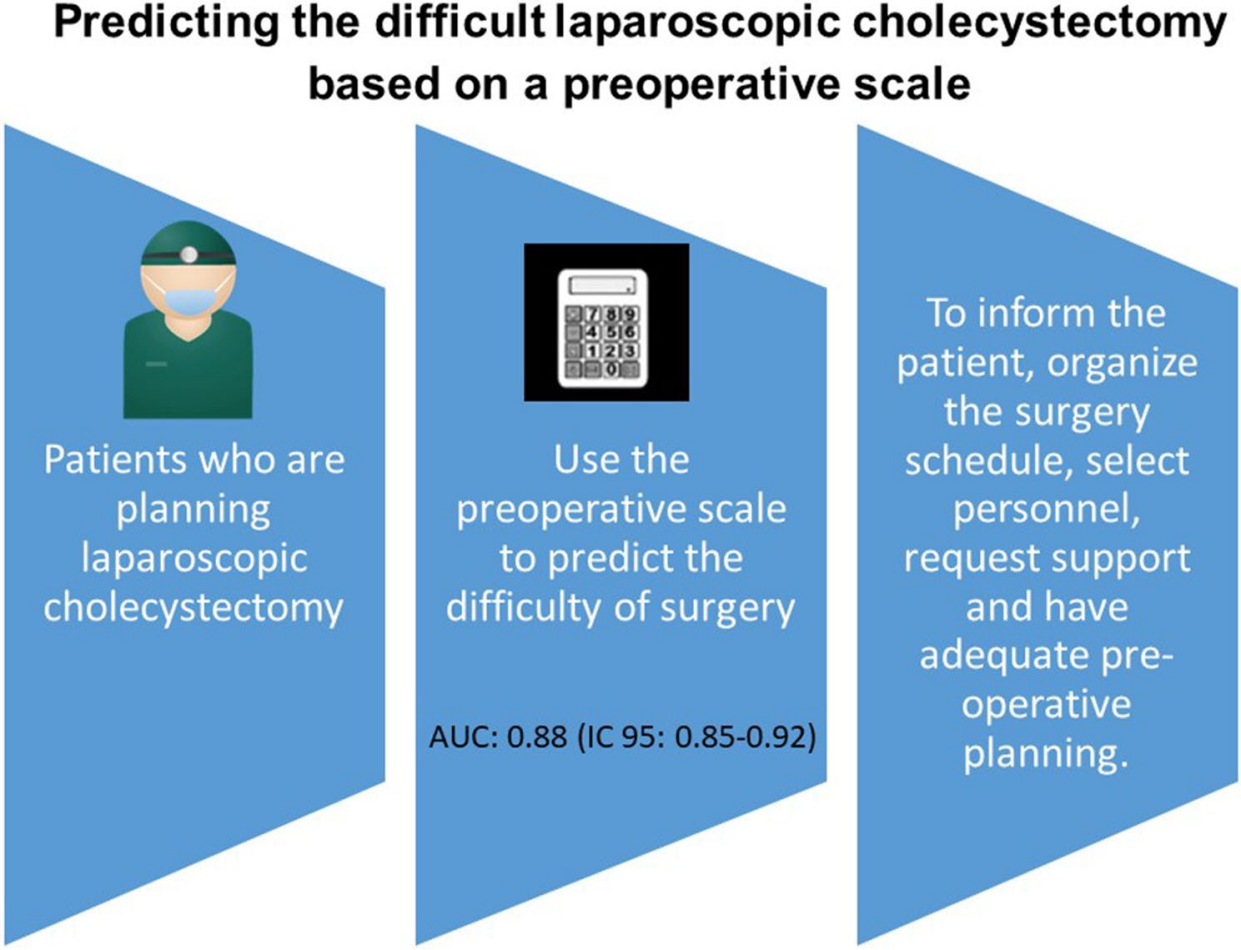

## Introduction

Laparoscopic cholecystectomy since its first description in 1985 has become the reference standard for the treatment of benign biliary disease. Nowadays it is one of the most frequently performed surgical procedures in the world, in our institution around 1500 cholecystectomies are performed annually [1, 2].

At the beginning of the development of the laparoscopic technique, there was a high rate of bile duct injuries and complications due to the learning curve, with time, serious lesions decreased from 0.08 to 0.12% and 1.5% of all lesions. The difficulty of cholecystectomy has been related to complications [1].

Multiple factors that may influence the difficulty of a cholecystectomy have been described, which may be related to the patient, such as age, sex, anatomical variations, previous surgeries, obesity, or may be related to pathologies such as severe inflammation or impacted stones, external factors such as failure of inappropriate equipment or equipment may also influence [1, 3–7].

The evaluation of this difficulty can also vary between the perception of a surgeon and another, hence the importance of using a single intraoperative difficulty scale, where intraoperative findings are described. To use one of these scales, it must be based on intraoperative findings and thus define the difficulty of laparoscopic cholecystectomy, which, regardless of the surgeon, will not change. Given the above, multiple scales such as Parkland, AAST, Cuschieri or Sugrue [8–10] have been described, another of these scales were described by Nassar et al., in 1995, which was recently validated in a study that included two prospective cohorts with a total of 12,909 patients. Intraoperative findings are standardized with the help of one of these scales [10].

The difficulty of the procedure can vary between one and the other, the key is to predict this difficulty. In the literature, we find multiple scales to predict a difficult cholecystectomy; however, most of these are based on the conversion rate or the surgical times, which can vary according to the experience of the surgeon [3, 4, 11, 12]. Therefore, these scales cannot be universally used.

The important thing about using the scales that predict the difficulty of cholecystectomy is that with this information we can choose the surgeon of the case, the schedule in which it is performed, optimize the pre-surgical planning and have an adequate informed consent for each patient to improve the outcomes [13].

Given the previously exposed drawbacks to predict a difficult cholecystectomy with an objective evaluation of this difficulty, Nassar et al., carried out a study where they developed and validated a system with preoperative variables to predict the difficulty of laparoscopic cholecystectomy, taking as reference standard the intraoperative scale described by this same author [13].

The aim of this study is to evaluate the predictive capacity of a difficult cholecystectomy with a preoperative scale described by Nassar at the Hospital Universitario Mayor Méderi in Bogotá, Colombia 2020.

It was decided to use the predictive and intraoperative scale described by Nassar because it is the only study found where this difficulty is evaluated with an objective intraoperative scale. Furthermore, the study was carried out in a population cohort with internal and external validity [10, 13].

## Methodology

A diagnostic trial study was designed to assess the performance of the scale for predicting the difficulty of laparoscopic cholecystectomy described by Nassar presented in Table 1 [10]; considering as a reference standard the intraoperative findings, evaluated according to the intraoperative difficulty scale described by the same author presented in Table 2 [13].

**Table 1:** Intraoperative difficulty scale for laparoscopic cholecystectomy [10]

Grade 1
Gallbladder—floppy, non-adherent
Cystic pedicle—thin and clear
Adhesions—simple up to the neck/Hartmann´s pouch
Grade 2
Gallbladder—mucocele, packed with stones
Cystic pedicle—fat laden
Adhesions—simple up to the body
Grade 3
Gallbladder—deep fossa, acute cholecystitis, contracted, fibrosis, Hartmann’s adherent to common bile duct, impaction
Cystic pedicle—abnormal anatomy or cystic duct—short, dilated or obscured
Adhesions—dense up to fundus; involving hepatic flexure or duodenum
Grade 4
Gallbladder—completely obscured, empyema, gangrene, mass
Cystic pedicle—impossible to clarify
Adhesions—dense, fibrosis, wrapping the gallbladder, duodenum or hepatic flexure difficult to separate

Easy: 1–2, difficult: 3–4

**Table 2:** Preoperative risk scale for difficult laparoscopic cholecystectomy [13]

Variable	Points
Age (years)	
< 40	0
40 +	1
Gender	
Female	0
Male	1
ASA classification	
1	0
2	1
3	2
4	7
Primary diagnosis	
Pancreatitis	0
Biliary colic	0
Choledocholithiasis	1
Cholecystitis	4
Thick-walled gallbladder (≥ 3 mm)	
No	0
Yes	2
Common biliary duct dilation (> 6 mm)	
No	0
Yes	1
Pre-operative ERCP	
No	0
Yes	1
Type of admission	
Elective	0
Delayed	1
Emergency	2

Low risk: 0–1, intermediate risk: 2–6, high risk: 7–19

**Fig. 1:**
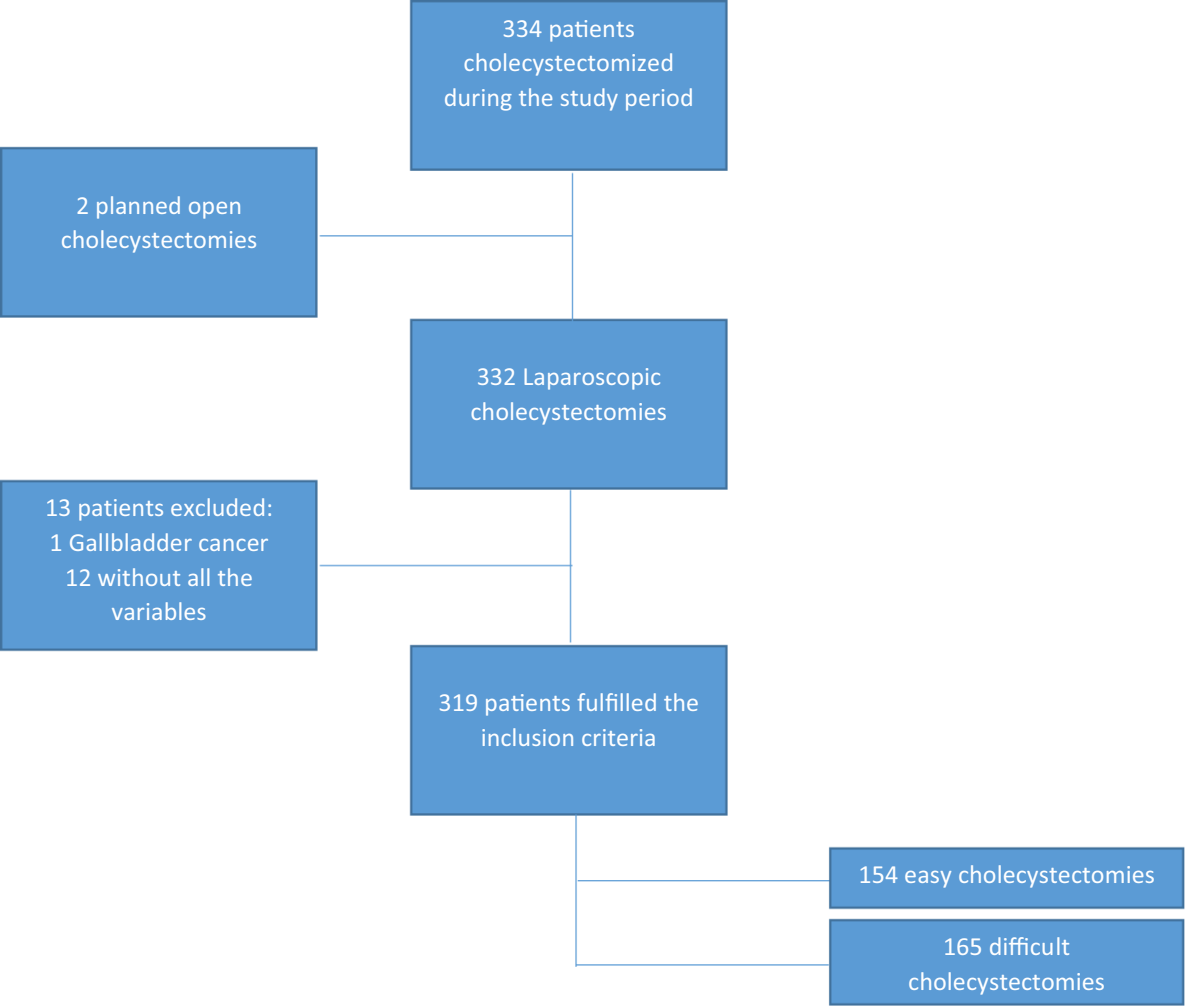
Flowchart of the study selection process

The medical history of patients over 18 years old, who underwent cholecystectomy between February and June 2020 were reviewed. Those medical records of the patients who complied with the protocol of the institution where the intraoperative difficulty was recorded in the medical history and documented by means of a photographic record were collected in anonymous database were selected for the study. The variables were collected in an anonymous database. Patients with planned open cholecystectomy, with gallbladder cancer and those who did not have all the variables necessary to calculate the risk of preoperative difficulty were excluded from the study (see Fig. 1).

The indication for cholecystectomy in all cases was a benign biliary disease, which had at least one diagnostic image (ultrasound, magnetic resonance cholangiopancreatography or tomography). The diagnosis of cholecystitis was made according to the Tokyo Guidelines [14]. As a protocol according to the risk of choledocholithiasis based on the American guidelines for choledocholithiasis, it was defined to perform cholecystectomy without additional studies, magnetic resonance cholangiopancreatography or endoscopic retrograde cholangiopancreatography (ERCP) [15].

Before initiating the dissection of the gallbladder, a photographic registry of the gallbladder was made, in addition to a record in the medical history, and intraoperative difficulty was classified from 1 to 4 according with the scale described by Nassar, considered the reference standard (Table 1).

The preoperative difficulty scale was calculated for each patient based on the pre-operative risk scale described by Nassar (Table 2), taking into account the patient's clinical history and diagnostic imaging studies.

Due to the retrospective design of the study, the surgeon who performed the cholecystectomy and described the intraoperative difficulty did not know the result of the preoperative scale, which was calculated subsequently by the researchers.

The study was performed according to the list of essential items reporting diagnostic accuracy studies [16].

This study did not represent any intervention on the patients and all the information was collected retrospectively from their medical records. For this reason, it is considered at risk-free study according to the Colombian law. The confidentiality of individual data was preserved. Upon admission to the institution, patients gave a written informed consent to use their clinical information for research purposes. The study protocol and statistical analysis was approved by the research committee of the Hospital Universitario Mayor—Méderi and by the ethics committee of Universidad del Rosario (number DVO005 1404-CV1301).

### Statistical analysis

A description of the demographic variables, risk factors and surgical outcomes was made. Categorical variables were described in proportions and continuous variables in means. It was assessed whether there were statistically significant differences (*p* < 0.05) between the variables of factors associated with difficult cholecystectomy.

A ROC curve was performed and used to estimate the diagnostic and predictive value of the preoperative score to predict intraoperative findings. The sensitivity, specificity, predictive values, and Youden index were calculated for the different cut-off points on the preoperative scale.

The sample was calculated for an expected sensitivity of 93.4%, a difficult cholecystectomy prevalence of 31.2%, a 95% confidence interval and 5% accuracy while also estimating a loss of 10%. This expected sensitivity and prevalence were taken from the previous study of Nassar et al. [13].

The entire analysis was performed in Epidat 4.2, considering a statistically significant *p* < 0.05.

## Results

A total of 319 patients were included in the study, a flowchart shows the selection process (Fig. 1). Two open planned cholecystectomies were performed, one due to septic shock and one given multiple supraumbilical surgical history. Another 12 patients were excluded because they did not have all the variables to calculate the preoperative risk and one remaining the pathology showed gallbladder cancer. Therefore, 319 met the inclusion criteria and were selected for analysis.

**Fig. 2:**
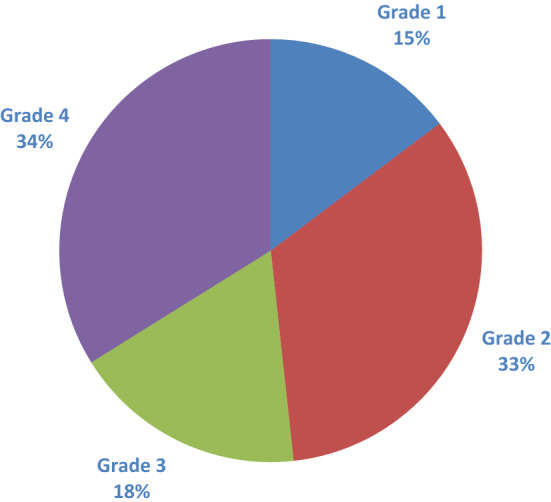
Distribution of difficulty according to The Nassar scale

The evaluated patients had an average age of 55.4 $\pm$ 18.2 and there was a female predominance (57.99%), others demographic characteristics can be observed in Table 3. The degree of difficulty evidenced by the surgeon in the reported intraoperative can be observed in Fig. 2, recalling that grades 1–2 are classified as easy and 2–3 as difficult.

**Table 3:** Demographic characteristics

	*N* (%)
Age (mean ± SD) (years)	55.4 ± 18.2
Sex	
Female	185 (57.99)
Male	134 (42.01)
ASA classification	
1	109 (34.17)
2	138 (43.26)
3	71 (22.26)
4–5	1 (0.31)
Primary diagnosis	
Pancreatitis	8 (2.51)
Biliary colic	132 (41.38)
Choledocholithiasis	37 (11.6)
Cholecystitis	142 (44.51)
Gallbladder wall thickness (mean ± SD) (mm)	3.3 ± 1.7
Diameter common bile duct (mean ± SD) (mm)	4.9 ± 2.1
Pre-operative ERCP	
No	256 (80.25)
Yes	63 (19.75)
Type of admission	
Elective	58 (18.18)
Delayed	252 (79.00)
Emergency	9 (2.82)

In the bivariate analysis of the variables that includes the pre-operative scale, we found that it is more likely to find a higher intraoperative degree of difficulty with an age greater than 40 years, a higher ASA classification, the type of admission, a gallbladder walls greater than or equal to 3 mm, a bile duct greater than or equal to 6 mm and a diagnosis of cholecystitis. However, no statistically significant difference (p 0.467) is found in terms of the pre-operative ERCP (Table 4).

**Table 4:** Comparison of pre-operative factors between an easy and a difficult cholecystectomy

	Easy (%) 154 (48.27)	Difficult (%) 165 (51.72)	*p* value
Age (years)			** < 0.001**
< 40	53 (16.61)	22 (6.89)	
40 +	101 (31.66)	143 (44.82)	
Gender			** < 0.001**
Female	110 (34.48)	75 (23.51)	
Male	44 (13.79)	90 (28.21)	
ASA classification			< 0.001*
1	65 (20.37)	44 (13.79)	
2	75 (23.51)	63 (19.74)	
3	14 (4.38)	57 (17.86)	
4–5	0 (0)	1 (0.31)	
Primary diagnosis			
Pancreatitis	7 (2.19)	1 (0.31)	< 0.001*
Biliary colic	106 (33.22)	26 (8.15)	
Choledocholithiasis	22 (6.89)	15 (4.70)	
Cholecystitis	19 (5.95)	123 (38.55)	
Thick-walled gallbladder (≥ 3 mm)			** < 0.001**
No	121 (37.93)	35 (10.97)	
Yes	33 (10.34)	130 (40.75)	
Common bile duct dilation (> 6 mm)			
No	131 (41.06)	119 (37.30)	**0.005**
Yes	23 (7.21)	46 (14.42)	
Pre-operative ERCP			**0.467**
No	121 (37.93)	135 (42.31)	
Yes	33 (10.34)	30 (9.40)	
Type of admission			< 0.001*
Elective	52 (16.30)	6 (1.88)	
Delayed	102 (31.97)	150 (47.02)	
Emergency	0 (0)	9 (2.82)	
Preoperative risk of difficult laparoscopic cholecystectomy			< 0.001*
Low (0–1)	32 (10.03)	2 (0.62)	
Intermediate (2–6)	94 (29.46)	29 (9.09)	
High (≥ 7)	28 (8.77)	134 (42)	

The *p* values were obtained from the chi-square test

Bold values indicate statistically significant *p* values (*p* < 0.05)

*The *p* values were obtained from the Mann–Whitney test

As for the risk of difficult cholecystectomy calculated with the preoperative scale, we found a difference (p < 0.001) between the proportions of the agreement if the risk is low, intermediate or high (Table 4).

**Fig. 3:**
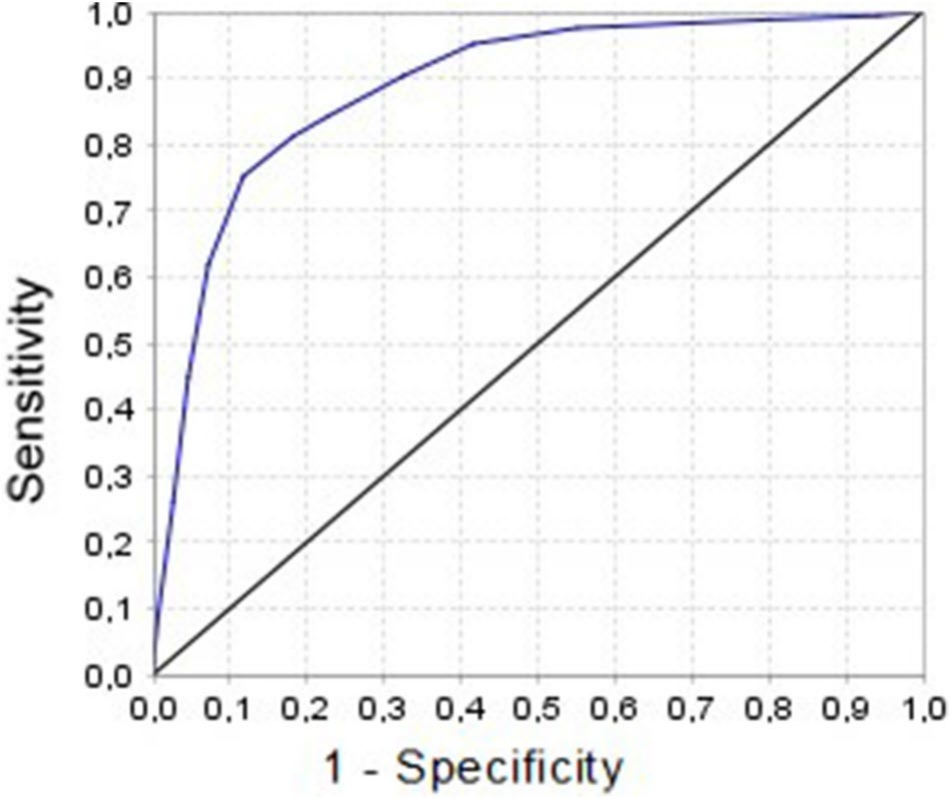
ROC curve

The ROC curve shows an area of 0.88 under the curve (IC 95: 0.85–0.92). We calculated for each cut-off point of the preoperative scale which was the performance to predict a difficult cholecystectomy according to the intraoperative scale (reference standard) (Fig. 3 and Table 5).

**Table 5:** Predictability of difficult cholecystectomy at different cut-off points

Cut-off point	Sensitivity	Specificity	Predictive positive value	Predictive negative value	Youden’s index
1	0.99	0.05	0.53	0.90	0.04
2	0.98	0.20	0.57	0.94	0.18
3	0.97	0.44	0.65	0.94	0.41
4	0.95	0.58	0.71	0.91	0.53
5	0.90	0.67	0.74	0.86	0.57
6	0.84	0.77	0.79	0.82	0.61
7	0.81	0.81	0.82	0.82	0.62
8	0.75	0.88	0.87	0.76	0.63
9	0.61	0.92	0.90	0.69	0.53
10	0.44	0.95	0.91	0.61	0.39
11	0.26	0.97	0.91	0.55	0.23
12	0.09	0.99	0.93	0.50	0.08
13	0.01	1.00	1.00	0.48	0.01

No patients had a score greater than or equal to 14

Finally, the conversion rate, subtotal cholecystectomy rate, complication rate (bleeding and bile duct injury), and critical view of safety failed rate were evaluated according to the risk of difficult cholecystectomy calculated preoperatively (Table 6).

**Table 6:** Surgical results according to the risk of difficult cholecystectomy calculated preoperatively

	Low risk *N* = 34 (%)	Intermediate risk *N* = 123 (%)	High risk *N* = 162 (%)
Conversion from laparoscopic to open	0 (0)	2 (1.62)	13 (8.02)
Subtotal cholecystectomy	0 (0)	4 (3.25)	14 (8.64)
Critical view of safety failed	1 (2.94)	7 (5.69)	24 (14.81)
Complications			
Bleeding	0 (0)	2 (1.62)	4 (2.46)
Bile leak	0 (0)	0 (0)	2 (1.23)
Common bile duct injury	0 (0)	0 (0)	1 (0.61)

Low risk: 0–1, intermediate risk: 2–6, and high risk: 7–19

## Discussion

Laparoscopy cholecystectomy is one of the most common procedures in the world. During surgeon training, this procedure is the initial procedure when you begin your training in laparoscopy; however, in some cases may be technically difficult due to the inflammatory process and adhesions [4, 17].

When a difficult cholecystectomy occurs, the risk of bile duct injury increases by up to 10 times, increases the conversion rate, increased bleeding, more postoperative complications and longer surgical time [4, 7, 18].

It is important to have a tool to predict the difficulty of cholecystectomy, this to choose the best schedule to perform the procedure, have support, inform the patient of the possible difficulty and increase of complications, and select the patient for the patient's training according to the level of training [4, 13]. Risk factors have been established and evaluated at different predictor scales, however, these predictor scales predict conversion rate or surgical time, which are not objective measures because they will depend on surgeon training [13].

To evaluate the diagnostic performance of a predictor scale having as a reference standard a validated intraoperative scale, it was decided to carry out this study. The predictability of the presurgical scale is adequate, with an area under the curve better than the one reported in the study in which it was carried out. The cut-off point where it has the highest performance to predict a difficult cholecystectomy is 8 with the highest Youden index.

Within the variables that include scale all except pre-operative ERCP were associated with a difficult cholecystectomy. This may be because it was only performed on 63 patients in our series.

It should be noted that the proportion of difficult cholecystectomies performed in our environment was 51.72% while in the index study it was between 29.4% and 35.1%, which may be secondary possibly to a lower rate of elective procedures, a health system where the surgical opportunity is later and a high rate of cholecystitis.

The intraoperative scale does predict complications such as bile duct injury, intestinal injury, bleeding, among others; in addition to predicting conversion from laparoscopic to open cholecystectomy [10]. In other words, by using preoperative predictive scale, predicting the degree of intraoperative difficulty, we are predicting the risk of complications, conversion from laparoscopic to open, subtotal cholecystectomy and critical view of safety failed [10, 19]. We can observe in our results, as the predicted difficulty increases from low risk to intermediate risk to high risk, the rate of conversion to open procedure, the rate of subtotal cholecystectomies, the rate of complications and the rate of critical view of safety increase.

If a high preoperative risk of difficult cholecystectomy is predicted, which implies a greater possibility of elevated intraoperative difficulty, it would be a discretionary decision of the surgeon, to define whether to avoid complications it is better to perform non-operative management or with cholecystostomy.

Among the limitations of the study are the subjectivity of the intraoperative scale which was controlled with the photographic record, its retrospective nature, and the fact that we only included the variables described in the preoperative scale, therefore, other variables that can be related to the difficulty such as obesity, surgical history, leukocytosis, among others, were not evaluated.

In conclusion, we suggest implementing the preoperative scale described by Nassar et al., in all patients who are planning laparoscopic cholecystectomy, considering it a simple and easy-to-use tool. This in order to inform the patient, organize the surgery schedule, select personnel, request support and have adequate pre-operative planning.

## Data Availability

Data are available on request through institutional review board of Hospital Universitario Mayor—Méderi. You can contact to request the data to jose.daza@mederi.com.co.
